# Functionalisation of Virus-Like Particles Enhances Antitumour Immune Responses

**DOI:** 10.1155/2019/5364632

**Published:** 2019-01-08

**Authors:** Katrin Kramer, Farah Al-Barwani, Margaret A. Baird, Vivienne L. Young, David S. Larsen, Vernon K. Ward, Sarah L. Young

**Affiliations:** ^1^Department of Pathology, Dunedin School of Medicine, University of Otago, Dunedin, New Zealand; ^2^Biology Department, Sultan Qaboos University, Muscat, Oman; ^3^Department of Microbiology and Immunology, School of Biomedical Sciences, University of Otago, Dunedin, New Zealand; ^4^Department of Chemistry, Division of Sciences, University of Otago, Dunedin, New Zealand

## Abstract

Virus-like particles (VLP) from the rabbit haemorrhagic disease virus (RHDV) can deliver tumour antigens to induce anticancer immune responses. In this study, we explored how RHDV VLP can be functionalised to enhance the immune response by increasing antigen loading, incorporating linkers to enhance epitope processing, and targeting receptor-mediated internalisation of VLP. RHDV VLP were developed to deliver up to three copies of gp100_25–33_ which contained proteasome cleavable linkers to target the correct processing of the epitope. Addition of mono- and dimannosides, conjugated to the surface of the gp100 VLP, would utilise a second pathway of internalisation, mannose receptor mediated, to further augment antigen internalised by phagocytosis/macropinocytosis. *In vitro* cell culture studies showed that a processing linker at the C-terminus of the epitope (gp100.1LC) induced enhanced T-cell activation (7.3 ng/ml interferon- (IFN-) *γ* release) compared to no linker (3.0 ng/ml IFN-*γ*) or the linker at the N-terminus (0.8 ng/ml IFN-*γ*). VLP delivering two (gp100.2L) or three (gp100.3L) gp100 epitopes induced similar high T-cell activation (7.6 ng/ml IFN-*γ*) compared to gp100.1LC. An *in vivo* cytotoxicity assay and a therapeutic tumour trial confirmed that mice vaccinated with either gp100.2L or gp100.3L induced a specific antitumour immune response. Mannosylation of the gp100.2L VLP further enhanced the generated immune response, demonstrated by prolonged survival of mice vaccinated with dimannosylated gp100.2L VLP (D-gp100.2L) by 22 days compared to gp100.2L-vaccinated mice. This study showed that functionalisation of RHDV VLP by addition of an epitope-processing linker and mannosylation of the surface facilitates the efficacy of VLP as vaccination vectors for tumour immunotherapy.

## 1. Introduction

Virus-like particles (VLP), nanoparticles that self-assemble from capsid proteins of viruses, are stable, highly immunogenic, and proven safe to use in vaccine formulations [1, 2]. While VLP can be used to provide protection against the parent virus, they can also be exploited as a vaccine platform against a variety of diseases by delivering foreign antigens [3, 4]. Importantly, VLP acquired by antigen-presenting cells (APC), such as dendritic cells (DC), can induce a cellular antitumour immune response, which can be utilized for cancer immunotherapy.

This study used VLP derived from the rabbit haemorrhagic disease virus (RHDV) which is composed of 180 copies of the viral capsid protein VP60 [3]. As the parental virus is not human derived, it avoids preexisting immunity to the virus which makes it suitable for use in immunotherapy. Importantly, the RHDV VLP can be engineered to recombinantly express repetitive tumour epitopes, such as the glycoprotein 100 (gp100) peptide KVPRNQDWL (gp100_25–33_). gp100 is a widely studied melanoma-associated antigen that is involved in melanosome maturation and melanin synthesis [5]. The gp100_25–33_ peptide used in this study is an H-2D^b^ restricted epitope that has shown promise in tumour immunotherapeutic trials and is used as an established murine melanoma model [6, 7]. We have shown previously that RHDV VLP delivering tumour antigens are immunogenic and are capable of inducing a strong immune response [4, 8, 9].

Cancer vaccination using RHDV VLP which delivers tumour antigens involves the initiation of a cellular immune response against the specific tumour epitopes. The VLP is first internalised by APCs via phagocytosis, macropinocytosis, or receptor-mediated endocytosis [10, 11]. Antigens acquired externally are usually processed onto major histocompatibility complex- (MHC-) II; however, we have shown that tumour antigens delivered by RHDV VLP can be cross-presented onto MHC-I [10]. Following cross-presentation, naïve T lymphocytes bind to the antigen epitope presented on MHC-I as well as costimulatory molecules upregulated on APCs. This can activate both CD8+ cytotoxic T lymphocytes (CTL) and CD4+ T-helper cells (Th) for a strong, cell-mediated cytotoxic immune response [12].

RHDV VLP are a versatile delivery platform capable of generating innate and adaptive immune responses [4, 13]. In this study, we explore how functionalisation of gp100-delivering RHDV VLP can enhance the specific anticancer immune response. Several key stages of the immune response initiation can be targeted to enhance the VLP efficacy: (i) enhancing the total number of epitopes presented per VLP unit, (ii) enhancing the processing of presented epitopes by the APC, and (iii) functionalisation of the VLP to enhance uptake by APCs. Here, we report that incorporating more gp100 antigen per RHDV VLP subunit (one, two, and three copies) did not affect the activation of T-cells *in vivo*. To enhance processing of the gp100 epitope, cleavable processing linkers between the VLP subunit and the gp100 epitope were added which enhanced T-cell activation *in vitro*. Our group previously reported that mannosylation of the surface of VLP enhances uptake of the VLP by APCs via the mannose receptor [14]. In this study, we demonstrate that the enhanced uptake of VLP translates into superior survival of mice vaccinated with mannosylated VLP in a melanoma tumour trial.

## 2. Materials and Methods

### 2.1. RHDV VLP Expression

RHDV VLP containing the various gp100_(25–33)_ epitopes were produced in insect cells by recombinant baculovirus expression. Briefly, the various gp100 DNA sequences were either PCR amplified or commercially synthesised (Genscript) and individually cloned onto the N-terminus of the RHDV VP60 gene, under the control of the p10 promoter. The recombinant baculoviruses were generated by homologous recombination and each gp100.VLP was expressed and purified as previously described ([13]). For each VLP, the presence of the epitope was confirmed by mass spectrometry at the Otago Centre for Protein Research and assembly was confirmed by transmission electron microscopy at the Otago Centre for Electron Microscopy.

### 2.2. Mannosylation of VLP

Chemical synthesis of N-succinimidyl- (NHS-) activated mannosides has been described previously [14]. To conjugate the NHS-mannosides to VLP, RHDV VLP in PBS was conjugated to a 50-fold molar excess of the mannosides for 3 h at room temperature and then overnight at 4°C. Following mannosylation, free mannosides were removed by dialysis and conjugation was confirmed by mass spectrometry. Quantification of mannosylation was estimated by lectin blot and Carbohydrate Estimation Kit (Thermo Scientific).

### 2.3. Animals: Source and Ethics

Specific-pathogen-free C57BL/6 mice were sourced from the Hercus Taieri Research Unit, University of Otago, Dunedin, New Zealand. Pmel mice (B6.Cg-Thy1a/Cy Tg(TcraTcrb)8Rest/J) were obtained from The Jackson Laboratory (Bar Harbour, ME, USA) and the colony maintained in house. The mice were genotyped by standard polymerase chain reaction (PCR) using DNA isolated from ear notches with the following primers: TCR *α* forward, 5′-GGT CCT GTG GCT CCA GTT TAA T-3′; TCR *α* reverse, 5′-CTG CTT AAC CTG TCC CTC ATG T-3′; TCR *β* forward, 5′-CTG GGC AGT GTT CTG TCT CC-3′; and TCR *β* reverse, 5′-ACC ATG GTC ATC CAA CAC AG-3′ [15]. The DNA amplification products were analysed on a 1% agarose gel. Breeding and experiments were conducted in accordance with ethical permits granted by the University of Otago Animal Ethics Committee (AUP 17/25, ET 22/11, ET 10/13, ET 17/17, AEC 97/13, and AEC 13/14). All animals were euthanized by cervical dislocation or carbon dioxide euthanasia.

### 2.4. Generation of Bone Marrow-Derived Dendritic Cells

Bone marrow-derived dendritic cells (BMDC) from C57Bl/6 mice were prepared as described by Inaba et al. [16]. Briefly, femurs and tibiae from euthanized mice were isolated and the red blood cells were lysed with ammonium chloride. The remaining cells were cultured in complete Iscove's Modified Dulbecco's Medium (cIMDM) containing 5% heat-inactivated foetal calf serum (FCS) and 20 ng/ml murine granulocyte/macrophage colony-stimulating factor (mGM-CSF) (R&D systems). On day six, BMDCs (5×10^4^ cells/ml) were pulsed for 24 h with either PBS, a VLP (10 *μ*g/ml), or gp100_25–33_ peptide (0.38 *μ*g/ml, molar equivalent to gp100 peptide in VLP-2L-gp100).

### 2.5. BMDC—T-Cell Coculture for Proliferation

Ammonium chloride-treated splenocytes from Pmel mice were sorted for CD8*α* T-cells (MicroBeads, Miltenyi, Bergisch Gladbach, Germany), stained with carboxyfluorescein succinimidyl ester (CFSE) (Invitrogen) (20 *μ*M), and added to BMDCs at a BMDC : T-cell ratio of 1 : 10. After 72 h, cells were stained with LIVE/DEAD® Fixable Near-IR Dead Cell Stain, treated with CD16/CD32 Fc blocking antibody, and then stained with CD3-PE-CF594 (clone 145-2C11) and CD8*α*-APC (clone 53-6.7). Fluorescence was measured using a Gallios flow cytometer (Beckman Coulter, Brea, CA, USA) with a three-laser (405 nm, 488 nm, and 633 nm), ten-colour configuration and analysed using Kaluza software (Beckman Coulter, Brea, CA, USA). Statistical analysis of the results was performed using GraphPad Prism version 6.0b.

### 2.6. BMDC—T-Cell Coculture for Interferon-*γ* Production

Ammonium chloride-treated splenocytes from Pmel mice were added to BMDCs at a BMDC : T-cell ratio of 1 : 10. After 72 h, the cell culture supernatants (150 *μ*l) were harvested and IFN-*γ* levels were measured by enzyme-linked immunosorbent assay.

### 2.7. In Vivo Cytotoxicity

Cytotoxicity was established as described previously [4, 8, 9]. In brief, groups of 6 female C57BL/6 mice aged 7–9 weeks were vaccinated with cPBS (0.03 M NaH2PO4.2H2O, 0.17 M Na2HPO4, and 0.15 M NaCl, pH 7.4), 100 *μ*g of VLP in cPBS, or equimolar quantities of synthetic gp100_25–33_ (KVPRNQDWL) in cPBS, combined with 25 *μ*g Cpg 1826 (GeneWorks, SA, Australia). Each treatment was administered subcutaneously into the left flank in 100 *μ*l solution. After 21 days, a boost of the same treatment was given to each mouse. Target cells were prepared from C57Bl/6 donor mice splenocytes. Ammonium chloride-treated cells were separated into three populations: unpulsed, pulsed with 10 *μ*M of human gp100 epitope (KVPRNQDWL), or murine gp100 epitope (EGSRNQDWL). Following 2 h incubation at 37°C, 5% CO_2_, the populations were stained with one of the following dye combinations: 0.1 *μ*M CFSE (CFSE^Lo^), 2 *μ*M CFSE (CFSE^Hi^), or 4 *μ*M violet proliferation dye (VPD). Target cells were injected intravenously into the mice. After 40 h, spleens from vaccinated mice were harvested, ammonium chloride treated, and lymphocytes stained with LIVE/DEAD Fixable Near-IR Dead Cell Stain. Fluorescence was measured using a Gallios flow cytometer and analysed using Kaluza. Statistical analysis of the results was performed using GraphPad Prism version 6.0b.

### 2.8. Therapeutic Tumour Trial

B16.gp33 cells were injected s.c. into the left flank of age-matched CL57BL/6 mice (5 × 10^5^ cells/mouse, *n* = 5 mice per treatment). When tumours were palpable on day seven, mice were injected with cPBS or 100 *μ*g of VLP in cPBS, combined with 25 *μ*g Cpg 1826. Tumour size was measured every two days, and when the tumour reached 150 mm^2^, mice were culled by cervical dislocation. Statistical analysis of the results was performed using GraphPad Prism version 6.0b.

## 3. Results

### 3.1. VLP and Linker Design

**Figure 1:**
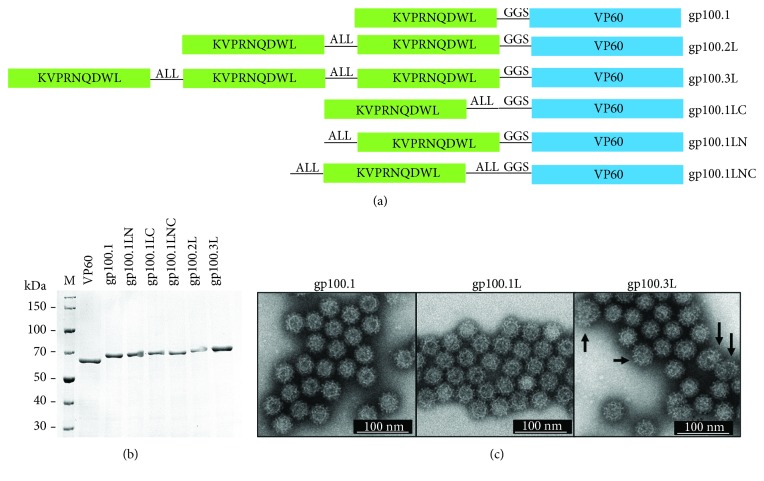
gp100 RHDV VLP design and expression. Each gp100 RHDV VLP was expressed in Sf21 cells and purified for analysis and applications in vaccination. (a) Diagram outlining the structure and linker position for each gp100 VLP. (b) SDS-PAGE for RHDV VLP with one (gp100.1, 1LN, 1LC and 1LNC), two (gp100.2L), and three (gp100.3L) gp100 epitopes compared to unmodified VP60. (c) Electron microscopy to confirm particle formation for gp100.1, 2L, and 3L.

Substituting the first three amino acids from the murine gp100_25–33_ epitope EGSRNQDWL to the human gp100_25–33_ epitope KVPRNQDWL enhances its binding to H-2D^b^ [17, 18]. This was confirmed by testing both the human and mouse sequences with IEDB MHC-I binding prediction tools. Human gp100_25–33_ was found to have a slightly lower percentile rank (4) than murine gp100_25–33_ (4.2), indicating enhanced binding to H-2D^b^. The human gp100_25–33_ epitope was therefore selected as target antigen for this study. The RHDV VLP were designed to express either one, two, or three copies of gp100_25–33_ (Figure 1(a)). Multiple short linker sequences (glycine-glycine-serine (GGS) [19], alanine-leucine-leucine (ALL) [20], alanine-alanine-tyrosine (AAY) [20], serine-serine-leucine (SSL) [20], glycine-valine-alanine-threonine (GVAT) [21], and glycine-threonine (GT) [21]) were entered into the proteasome and immunoproteasome prediction programs IEDB, PCPS, and NetChop and ranked based on the total number of cleavage sites, the number of desired cleavage sites, and the probability of cleavage at those sites. The prediction indicated that the GGS linker would not change the processing of the gp100 epitopes; it was selected to be incorporated before the VP60 gene as a flexible linker. ALL was ranked first across all prediction programs, indicating enhanced efficiency over the other tested sequences (Figure S1) and was therefore selected as a cleavable linker and incorporated in between the gp100_25–33_ copies, as detailed in Figure 1(a). Each RHDV gp100.VLP was expressed in insect cells using a recombinant baculovirus and purified by differential and CsCl gradient centrifugation (Figure S2). The VP60 capsid proteins spontaneously form particles of approximately 40 nm in diameter that possess a cog-like structure. Purification and assembly was confirmed by SDS-PAGE (Figure 1(b)) and electron microscopy (Figure 1(c)).

### 3.2. *In Vitro* T-Cell Activation

**Figure 2:**
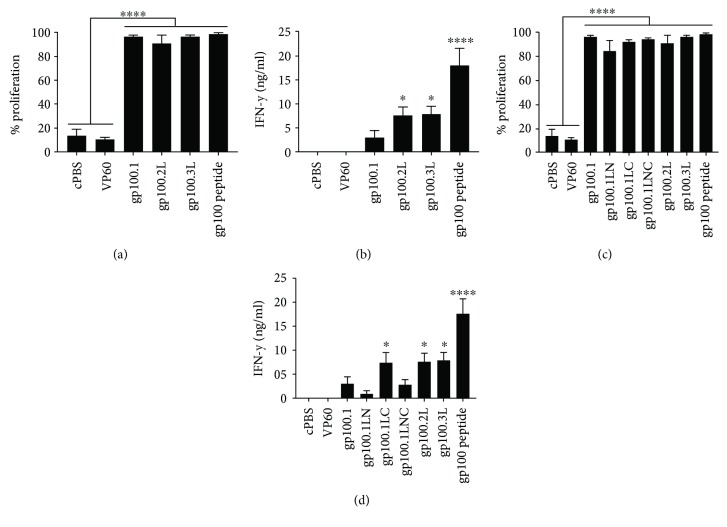
In vitro T-cell activation by gp100 VLPs. BMDC were pulsed with 10 *μ*g/ml VLP or gp100 peptide at a molar equivalent to gp100 in gp100.2L VLP. CD8+ T-cells from Pmel mice were cocultured with activated BMDC for 72 h. (a, c) Proliferation of T-cell in coculture, measured by flow cytometry. (b, d) Supernatants from cocultures were harvested and IFN-*γ* measured by ELISA. Graphs show the mean of three independent experiments ± SEM. Statistical significance was determined by one-way ANOVA with Tukey's post hoc tests, ^∗∗∗∗^ *p* < 0.0001, ^∗^ *p* < 0.05. Statistical significance displayed is the comparison between untreated cells (cPBS and VP60) and treated cells.

The gp100 VLPs were tested to determine whether the addition of multiple antigen epitopes induced an enhanced T-cell response. Gp100.1, gp100.2L, and gp100.3L were pulsed onto BMDCs for 24 h followed by addition of CD8+ T-cells from Pmel mice. Following 72 h of coculture, CD8+ T-cell activation was measured by proliferation and IFN-*γ* release. All three gp100 VLPs (gp100.1, 2L, and 3L) induced very strong T-cell proliferation of about 90–95% comparable to the gp100 peptide control, which was significantly higher than the VP60 and PBS control (Figure 2(a)).  Figure 2(b) shows that cells pulsed with gp100.2L and 3L VLPs induced significantly enhanced IFN-*γ* release of 7.5 ng/ml compared to only 2.5 ng/ml when pulsed with gp100.1 VLP. This suggests that there is an enhanced T-cell activation when multiple epitopes are present albeit there was no further enhancement between gp100.2L and 3L. The lack of further enhancement with gp100.3L could be due to the reduced stability of the gp100.3L particle containing a longer insert (see arrows, Figure 1(c)).

To determine whether the approximately threefold increase in IFN-*γ* by gp100.2L and gp100.3L was due to the delivery of multiple gp100 epitopes or due to the addition of a cleavable linker between the epitopes, recombinant VLP with only one gp100 epitope with the ALL linker either before and/or after the epitope were designed (see Figure 1(a)). Cells pulsed with gp100.1, gp100.1LC, gp100.1LN, and gp100.1LNC VLPs as well as the gp100.2L and 3L as comparison were analysed for CD8+ T-cell activation.  Figure 2(c) shows high T-cell proliferation (85–95%) when the gp100 epitope is present regardless of the number of copies or the orientation of the cleavable linker. Cells pulsed with gp100.1LC VLP produced approximately 7.5 ng/ml IFN-*γ* comparable to gp100.2L and 3L, which suggests the placement of the linker may be important (Figure 2(d)). Addition of the ALL linker on the N-terminus of the gp100 epitope in gp100.1LN and gp100.1LNC resulted in low IFN-*γ* production comparable to gp100.1, while addition of the linker solely at the C-terminus in gp100.1LC enhanced IFN-*γ* production. While there was no significant difference, the 0.8 ng/ml IFN-*γ* produced by gp100.1LN was lower than the 3.0 ng/ml IFN-*γ* produced by gp100.1 pulsed cells and the 2.8 ng/ml IFN-*γ* produced by gp100.1LNC was lower than the 7.3 ng/ml IFN-*γ* produced by gp100.1LC pulsed cells. This suggests that the orientation of the processing linker may be a determining factor in inducing an augmented T-cell response. Based on this data, the gp100.2L and gp100.3L VLPs were selected as models for further testing *in vivo* as these two constructs included multiple antigen epitopes and the ALL processing linker.

### 3.3. *In Vivo* Evaluation of gp100 VLPs

**Figure 3:**
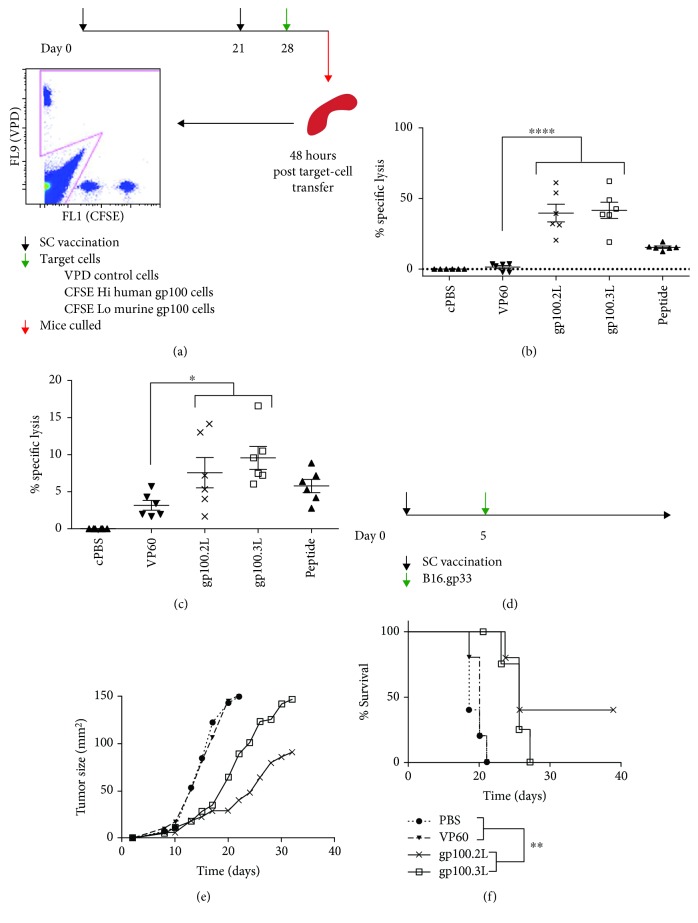
In vivo evaluation of gp100 VLPs. (a) Timeline of cytotoxicity assay. *n* = 6 mice were vaccinated on day 0 and 21 with 100 *μ*g VLP, cPBS negative control or gp100 peptide at a molar equivalent to gp100 within gp100.3L, each including 25 *μ*g CpG. On day 28, mice were injected with 1 × 10^7^ fluorescently labelled target cells, either pulsed with human gp100, murine gp100 peptide or left unpulsed. Mice were culled 48 hr after target cell injection, and target cells were analysed. Percentage of specific lysis of (b) human gp100 and (c) murine gp100 target cells normalised against the cPBS group. Statistical significance for cytotoxicity is the comparison between VP60- and gp100-vaccinated mice as determined by one-way ANOVA with Tukey's post hoc tests, ^∗∗∗^ *p* < 0.001, ^∗^ *p* < 0.05. (d) Vaccination strategy: *n* = 5 mice were injected s.c. with 5 × 10^5^ B16.gp33 tumour cells. On day 5, mice were vaccinated with 100 *μ*g VLP or cPBS negative control, each including 25 *μ*g CpG. The graphs show the (e) mean tumour growth rate or (f) survival proportions. Significance was determined using a log-rank (Mantel-Cox) test, ^∗∗^ *p* < 0.01.

Following enhanced T-cell activation and proliferation *in vitro*, the ability of gp100.2L and 3L VLPs to induce a target-specific cytotoxicity was assessed in an *in vivo* cytotoxicity assay. Mice were vaccinated subcutaneously with gp100 VLPs or controls, using CpG 1826 oligodeoxynucleotides (CpG) as an adjuvant, followed by a boost of the same treatment 21 days later. On day 28, mice were challenged with fluorophore-stained target cells pulsed with either the natural murine gp100 peptide or the human gp100 epitope included in the VLP vaccine as well as nonpulsed control cells (Figure 3(a)). Splenocytes were analysed 40 h after target cell challenge, and specific lysis (%) was calculated by comparing target and nontarget cell population. Figures 3(b) and 3(c) show that both gp100.2L and 3L induced target specific cytotoxicity against human gp100 (around 45%) and murine gp100 (around 10%). There was no difference observed between the gp100.2L and 3L VLP indicating both were equally efficient in inducing cytotoxic T-cells. The control peptide induced lower cytotoxicity for both the human and murine gp100, confirming that the delivery of peptide vaccines in a VLP leads to enhanced efficacy *in vivo*.

**Figure 4:**
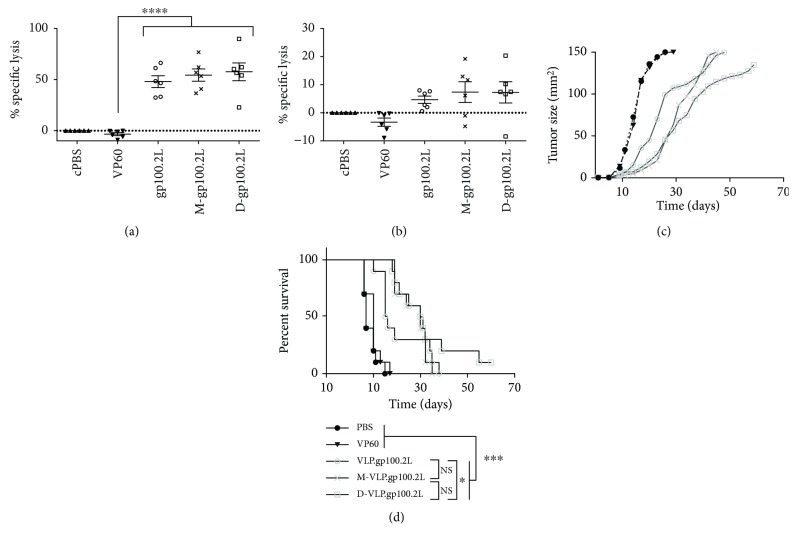
In vivo evaluation of mannosylated gp100 VLPs. *n* = 6 mice were vaccinated on day 0 and 21 with 100 *μ*g VLP or cPBS negative control, each including 25 *μ*g CpG. On day 28, mice were injected with 1 × 10^7^ fluorescently labelled target cells, either pulsed with human gp100, murine gp100 peptide or left unpulsed. Mice were culled 40 hr after target cell injection, and target cells were analysed. Percentage of specific lysis of (a) human gp100 and (b) murine gp100 target cells normalised against the cPBS group. Statistical significance displayed is the comparison between VP60- and gp100-vaccinated mice as determined by one-way ANOVA with Tukey's post hoc tests, ^∗∗∗^ *p* < 0.001. *n* = 10 mice were injected s.c. with 5 × 10^5^ B16.gp33 tumour cells. On day 5, mice were vaccinated with 100 *μ*g VLP or cPBS negative control, each including 25 *μ*g CpG. The graphs show the (c) mean tumour growth rate or (d) survival proportions. Significance was determined using a log-rank (Mantel-Cox) test, ^∗∗∗^ *p* < 0.001, ^∗^ *p* < 0.1.

The gp100.2L and 3L VLP were next tested in a therapeutic tumour trial to determine if they can provide protection against an established tumour. For this, mice were subcutaneously challenged with B16.gp33 tumour cells and vaccinated with the gp100 VLP and controls once the tumours were palpable on day 5 (Figure 4(d)). Mice vaccinated with gp100.2L and 3L all displayed delayed tumour growth compared to mice vaccinated with the PBS or VP60 controls. Addition of the third gp100 epitope in gp100.3L VLP provided no extra protection compared to mice vaccinated with gp100.2L. Conversely, mice vaccinated with gp100.2L showed the most delayed tumour growth (Figure 4(e)) as well as enhanced survival (Figure 4(f)), with two mice remaining tumour-free past day 60.

### 3.4. Mannosylation of VLP

Addition of mannose to the surface of VLP has been shown to enhance uptake of the VLP by APCs [14]. To determine whether enhanced uptake translates into altered efficacy *in vivo*, the surface of gp100.2L VLP was mannosylated with either a monomannose or dimannose as reported in detail by Al-Barwani et al. [14]. Mannosylated gp100.2L VLP were first assessed for their ability to generate a target-specific immune response in an *in vivo* cytotoxicity assay. Mice were subcutaneously vaccinated with gp100.2L, monomannose gp100.2L (M-gp100.2L), dimannose gp100.2L (D-gp100.2L), or controls using CpG as an adjuvant. On day 21, mice received the same treatments as a boost. As detailed above, mice were challenged with fluorescently labelled target cells (human gp100, murine gp100, or unpulsed) on day 28 and splenocytes were analysed for specific lysis 40 h after target cell challenge. While no statistically significant difference in the induced cytotoxicity could be detected between the VLPs, the mean lysis was higher for both the mono- and dimannosylated gp100.2L. The human gp100-specific lysis in response to VLP.gp100-2L increased by 6.4% following monomannosylation and 9.6% following dimannosylation (Figure 4(a)). As observed above, the specific cytotoxicity to gp100 VLPs was lower for murine gp100 target cells. While there was no statistical difference, mannosylation with either the monomannoside or the dimannoside lead to a 2.6% increase in cytotoxicity (Figure 4(b)).

As addition of mannosylation only showed a slight trend of enhancing the generated T-cell response to gp100.2L, the mannosylated VLPs were tested in a therapeutic tumour trial. B16.gp33 tumours were established in the right flank of mice, and once the tumours were palpable, mice were vaccinated with either gp100.2L, M-gp100.2L, D-gp100.2L, or controls, using CpG as adjuvant. All three gp100.2L VLPs significantly delayed tumour growth compared to the cPBS and VP60 controls (Figure 4(c)). While M-gp100.2L provided no enhancement of survival compared to the nonmannosylated gp100.2L VLP, dimannosylation significantly enhanced mouse survival compared to the nonmannosylated gp100.2L, with one mouse remaining tumour-free to day 70 (Figure 4(d)). Additionally, mice treated with M-gp100.2L and D-gp100.2L showed localised vitiligo at the site of vaccination, while mice vaccinated with gp100.2L without mannosylation did not demonstrate vitiligo (Figure S4). Taken together, this indicates that while both mono- and dimannosylation enhance uptake of VLP into APC (shown by Al-Barwani et al. [14]), only dimannosylation enhances antitumour T-cell responses *in vivo*.

## 4. Discussion

VLP induce robust CD4+ and CD8+ T-cell responses and have been shown effective in delivering tumour antigen peptides for immunotherapy [4, 8, 9]. Previous research demonstrated that VLP can be functionalised to enhance activation of innate immunity by incorporating adjuvants [13]. Conversely, this study aimed to enhance the adaptive immune response generated by VLP by improving antigen internalisation and processing by APC. The functionality of RHDV VLP was augmented by delivering multiple antigens, adding a processing linker around the antigen epitope and mannosylating the surface to enhance uptake by APCs.

Endogenous antigens are often not very immunogenic; however, substitution of single or multiple amino acids to slightly modify the sequence can lead to enhanced antigen binding to MHC molecules. This has been shown for multiple epitopes, including murine MUC1_1–8_ [22], human MART_26–35_ [23], and murine gp100_25–33_ epitopes [17, 18]. The human gp100 epitope was selected to be used as the tumour antigen for this research as the MHC-I prediction tools used in this study indicated enhanced binding to H-2D^b^ compared to the murine gp100_25–33_ epitope. First, the delivery of multiple antigen epitopes by the same VLP was tested as a potential functionalisation to the VLP. The N-terminus of the VLP capsid protein VP60 has been shown to support the insertion of up to 42 amino acids [24], thus the VLP were designed to either incorporate one, two, or three gp100_25–33_ epitopes. The second functionalisation was addition of processing linkers to the VLP. Linker sequences were added to separate the epitopes from both the VP60 and the other epitopes. In each recombinant VLP, the flexible linker GGS was incorporated between the VP60 gene and the antigen epitope to prevent the epitopes from hindering VLP assembly, as was reported previously [8, 14]. Additionally, a cleavable ALL linker [20] was added in between the gp100 epitopes to compare the importance of linker sequences in VLP processing and overall functionality. This ALL linker was aimed to target proteasomal cleavage during cross-presentation to increase the likelihood that the epitope is correctly cleaved during antigen processing.

Comparing the VLP with one, two, and three gp100 epitopes for their ability to stimulate T-cell activation showed that while gp100.1 did not enhance T-cell activation, both gp100.2L and 3L did to a similar extent. Availability of three gp100 epitopes as in gp100.3L did not increase the observed T-cell activation compared to having two epitopes; this may be explained by the reduced stability of the gp100.3L particles. However, it also posed the question whether the enhanced T-cell activation was due to the delivery of multiple gp100 epitopes or due to the ALL processing linker between the gp100 epitopes. To answer this, three more gp100 VLPs were made, each with a single gp100 epitope but with the ALL linker on either the gp100 C-terminus, the N-terminus, or on both termini. As the gp100.1LC VLP induced the same level of T-cell activation as the 2L and 3L VLP, the observed enhanced T-cell activation was due to the ALL processing linker. Although not statistically significant, addition of the ALL linker at the gp100 N-terminus seemed to decrease T-cell activation, as observed when comparing the IFN-*γ* levels produced by gp100.1 and 1LN as well as gp100.1LC and 1LNC. This may also explain why gp100.3L VLP did not induce further enhanced T-cell activation, as its second epitope, flanked by ALL linkers, may not be processed correctly. Having the linker on the N-terminus may therefore interfere with processing of the epitope to MHC-I. While the gp100 VLPs induced different levels of IFN-*γ* production, no difference in the proliferation of CD8+ T-cells was observed, as all gp100 VLPs induced very high proliferation of about 90%. A difference in proliferation may have been observed if a lower dose of VLP was used; however, the same dose of VLP was used to test both IFN-*γ* production and proliferation for consistency. Taken together, the *in vitro* data shows that a cleavable C-terminus is important for processing of the gp100 epitope and may also be important for other tumour antigen epitopes, albeit this was not tested here.

The gp100.2L and 3L VLPs were then tested for their ability to induce a targeted immune response *in vivo*, as these VLP encompass delivery of multiple antigen epitopes and include the ALL processing linker. While the cytotoxicity assay reflected the *in vitro* data, showing no difference in the specific lysis generated by either gp100.2L or 3L VLP, the gp100.2L VLP delayed tumour growth and induced enhanced survival in the therapeutic tumour trial.

In the therapeutic tumour trial, both gp100.2L and 3L induced enhanced survival compared to the controls, indicating that delivery of the gp100 epitope on VLPs can induce a gp100-specific immune response and substantial antitumour immunity. It also confirms that at least in this setting, the addition of a third copy of the same antigen provides no significant benefit to the functionality of RHDV VLP compared to two copies. Future work needs to address whether the three antigen epitope copies are processed correctly and whether the ALL linker around the second copy interferes with the proteasomal processing.

The third functionalisation of the gp100 VLP tested in this study was mannosylation of the VLP surface to enhance uptake by APCs. Previous work by our group showed that both mono- and dimannosylation of the surface of RHDV VLP significantly enhanced uptake of the VLP by both murine and human APCs [14]. Here, we showed that neither mono- nor dimannosylation of the gp100.2L VLP significantly enhanced specific lysis of target cells. However, when tested in a therapeutic tumour trial, dimannosylation of the gp100.2L VLP delayed tumour growth and thereby enhanced mouse survival. While the mice received two prophylactic vaccinations in the *in vivo* cytotoxicity experiment, the tumour trial only involved one therapeutic vaccination. The difference in response may be due to the vaccination schemes, it would therefore be interesting whether a suboptimal vaccination scheme in the cytotoxicity assay allowed a differentiation of the treatments. Furthermore, the vaccination in the therapeutic tumour trial relied on the fast generation of an immune response before the tumours grew to a size that is beyond the control of the immune system. The enhanced rate of uptake of the mannosylated VLP may therefore be more beneficial in a tumour setting as the control of the tumour depends on the rapid generation of an anticancer immune response. Additionally, receptors that show affinity for mannosylated ligands are known for preferentially binding to more complex mannosides [25–27], potentially explaining the enhanced survival of mice vaccinated with dimannosylated compared to manomannosylated gp100.2L. This response may be further enhanced by utilising a more complex mannoside which will be addressed in future studies. Interestingly, mice that were vaccinated with either the mono- or dimannosylated VLP demonstrated localised vitiligo at the site of vaccination. The vitiligo has been connected to an autoimmune destruction of melanocytes, with a number of groups shown that melanoma-associated vitiligo is often a favourable indication for melanoma patients [28]. Furthermore, enhanced vitiligo has been reported after administration of immunotherapies that drive T-cell responses to melanoma [29]. The observed vitiligo in the M-gp100.2L- and D-gp100.2L-vaccinated mice is therefore another indication of the ability of mannosylated VLP.gp100.2L to initiate enhanced gp100-specific immunity.

## 5. Conclusions

In conclusion, this work shows that functionalisation of VLP enhances a targeted antitumour immune response. Our data suggests that increasing the antigen loading on VLP does not affect the generated immune response. However, addition of a linker at the C-terminus of the antigen peptide facilitates epitope processing and thereby leads to an enhanced antitumour response. We additionally showed that targeting mannose-specific internalisation by mannosylating the VLP surface further enhances the generated immune response capable of significantly delaying the growth of murine melanoma. Combining these avenues of functionalisation identifies new strategies for improving tumour immunotherapies.

## Data Availability

The cell culture and in vivo data used to support the findings of this study are included within the article. Previously reported mannosylation data were used to support this study and are available at 10.1371/journal.pone.0104523. These prior studies (and datasets) are cited at relevant places within the text as a reference [14].

## Supplementary Materials

Supplementary 1 Figure S1: linker comparison in IEDB, PCPS, and NetChop prediction programs.

Supplementary 2 Figure S2: mass spectrometry results for gp100.1, gp100.2L, and gp100.3L VLPs.

Supplementary 3 Figure S3: gating strategy for T-cell proliferation: doublet cells were excluded by comparing forward scatter (FS) height with FS width and side scatter (SS) height with SS width. Live cells were gated as negative for near-infrared dye. Live cells were then gated on CD3+ and CD8+ cells followed by gating on proliferated CFSE stained cells.

Supplementary 4 Figure S4: vitiligo in tumour-free gp100 VLP treated mice: mice vaccinated with mannosylated gp100 VLP (a, b) had distinctive white patches at the site of vaccination (indicated with red circles).

Supplementary 5 Figure S5: in vivo cytotoxicity mouse weights: weights of mice injected with cPBS or gp100.2L VLP on day 0 and 21 of the in vivo cytotoxicity assay.
